# Gender-Oriented Mental Health Prevention: A Reappraisal

**DOI:** 10.3390/ijerph19031493

**Published:** 2022-01-28

**Authors:** Carla Comacchio, Giulia Antolini, Mirella Ruggeri, Marco Colizzi

**Affiliations:** 1 Azienda Ulss 9 Scaligera, 37126 Verona, Italy; carla.comacchio@univr.it; 2 Child and Adolescent Neuropsychiatry Unit, Maternal-Child Integrated Care Department, Integrated University Hospital of Verona, 37126 Verona, Italy; giuliaantolini11@gmail.com; 3 Section of Psychiatry, Department of Neurosciences, Biomedicine and Movement Sciences, University of Verona, 37134 Verona, Italy; mirella.ruggeri@univr.it; 4 Unit of Psychiatry, Department of Medicine (DAME), University of Udine, 33100 Udine, Italy; 5 Department of Psychosis Studies, Institute of Psychiatry, Psychology and Neuroscience, King’s College London, London SE5 8AF, UK

**Keywords:** gender, prevention, mental disorders, childhood abuse, pregnancy, intimate partner violence, education, employment, discrimination, relationships, healthcare systems

## Abstract

Many studies have investigated the impact of gender on mental health, but only a few have addressed gender differences in mental health risk and prevention. We conducted a narrative review to assess the current state of knowledge on gender-specific mental health preventive interventions, along with an analysis of gender-based risk factors and available screening strategies. Out of 1598 articles screened using a comprehensive electronic search of the PubMed, Web-of-Science, Scopus, and Cochrane databases, 53 were included for review. Among risk factors for mental health problems, there are individual, familiar, social, and healthcare factors. Individual factors include childhood adversities, which show gender differences in distribution rates. However, current childhood abuse prevention programs are not gender-specific. Familiar factors for mental health problems include maternity issues and intimate partner violence, and for both, some gender-specific preventive interventions are available. Social risk factors for mental health problems are related to education, employment, discrimination, and relationships. They all display gender differences, but these differences are rarely taken into account in mental health prevention programs. Lastly, despite gender differences in mental health service use being widely known, mental health services appear to be slow in developing strategies that guarantee equal access to care for all individuals.

## 1. Introduction

Mental disorders represent the fifth most common cause of disability worldwide [1]. Available treatment options do not result in a *restitutio ad integrum* for most mental conditions. Instead, investing efforts in mental health promotion, prevention, and early intervention may produce the best results [2]. The World Health Organization (WHO) recommends all neuropsychiatric interventions to be oriented towards promoting positive mental health at the population level, to enhance individuals’ abilities to monitor their mental health and its determinants [3]. On the other hand, prevention strategies aim to intervene in the pre-pathogenesis stage to prevent such conditions from arising whenever possible (primary prevention), promote early detection, screening, prompt treatment of disease, and limitation of disability (secondary prevention), and to sustain recovery, rehabilitation, and the restoration of functioning at the later stages of the disease (tertiary prevention) [4]. Thus, depending on the need of the individuals presenting at different stages in a continuum between mental health and disease [5], promotion and early intervention strategies aim at supporting the continuity of mental health care to achieve the best possible outcome in terms of wellbeing. Most prevention strategies so far have focused on psychosis, by applying an at-risk mental state (ARMS) concept implying the possibility to hamper the progression to full-blown disorder [6]. However, more recent evidence begins to support the possibility of widening the clinical area of intervention beyond psychosis, including common psychiatric disorders such as depression, anxiety, substance abuse, and eating disorders [7]. In this regard, an updated and more comprehensive clinical staging model has been proposed, the clinical high-risk mental state (CHARMS), which incorporates a trans-diagnostic paradigm [6]. A recent reappraisal of the literature suggests the importance of reorienting such interventions towards the youth population [8,9].

Oncology is one of the health sectors where prevention, early detection, multidisciplinary, and personalized strategies have led to tremendous advances, reshaping cancer care [10]. Lessons learnt from oncological research indicate that common risk factors for cancer diseases, such as alcohol, smoking, being overweight and physically inactive, have pro-cancer effects as a function of gender, with men and women being differentially exposed to the joint and multiplicative effects of risk factors [11]. Thus, despite all things being equal, loss of health is not equal among male and female individuals, urging the adoption of a gendered perspective for more effective public health interventions. 

Mental health is no exception. When developing preventive strategies for mental health, an important aspect to be considered is represented by gender, defined as socio-cultural constructs encompassing norms, rules, behaviors, and attitudes associated with being girls/women or boys/men, along with sex, which instead refers to a set of biological and physiological characteristics [12]. Gender interacts with differences between the sexes but represents a broader-spectrum dimension and may combine with other social factors, potentially resulting in a differential exacerbation of biological susceptibilities. Considering their differential and interacting impact on mental health, both gender- and sex-related differences should always be taken into consideration, starting from the implementation of promotion of the adoption of screening strategies to identify those people at risk of developing mental health problems, to prevent their onset or at least allow action before they worsen [8]. Furthermore, unique challenges, specific health issues, and health disparities may be faced by people with non-conforming gender, and a recent review of the literature highlights the importance of promoting gender diversity, to avoid a negative impact on mental health [13]. 

Several risk factors mapping on a pluripotent pathological trajectory for mental disorders have been identified [8]. They exert most of their influence on mental health from the prenatal period to early adulthood, with a less relevant modulation occurring until old age [14]. Additionally, pregnancy, parenthood, childhood care, behaviors exhibited in a relationship, and socio-occupational environments are privileged areas for interventions [14,15,16,17,18]. Research studies suggest a different response to life events as a function of gender [19,20].

The aim of this narrative review is threefold: (i) to update on the current evidence regarding the role of gender, sex, and gender diversity in conferring risk for mental health disorders; (ii) to analyze available screening tools that have been developed to detect subjects at potential risk for mental health problems, in a gendered perspective; and (iii) to review the state-of-the-art on the implementation of gender-oriented mental health prevention strategies.

## 2. Materials and Methods

The present literature review aims to provide a comprehensive overview of research evidence on gender-based risk factors for mental health, methodological applications for their detection, and the extent to which they have been targets for mental health prevention and early intervention strategies. In particular, the current work aims to emphasize the relationship between these components of gender-based mental care and the need for their integration as a roadmap to advance the field. Such implementation may offer new directions for clinical research into the full development of a gender-based model of mental healthcare focused on prevention. 

### 2.1. Search Strategy

A literature search was performed using electronic databases (PubMed, Web of Science, Scopus, Cochrane), by employing a combination of the following terms: “prevention”, “mental disorders”, “gender”, “sex”, “childhood abuse”, “pregnancy”, “intimate partner violence”, “employment”, “relationships” and “healthcare systems”, on 15 October 2021. No predefined time window or age range for the study search was adopted, to be the most inclusive as possible. Further research evidence, gathered outside of the search or identified through manual search of the reference section of the included articles, was reported if considered appropriate by the authors. Publication data screening and extraction were performed following a 2-step selection process (conventional double-screening) conducted by 2 reviewers independently of each other (CC and GA). In the rare instances of discrepant screening, a consensus was reached through discussion with a third senior clinical researcher (MC).

### 2.2. Eligibility Criteria 

Studies were eligible for inclusion in this review if they assessed the risk factors or preventive strategies for mental health from a gendered perspective. Only original papers published in English in peer-reviewed journals were accepted for inclusion in this review. By using a three-step screening approach, articles were screened through title, abstract, and full-text reading, if needed. Studies were excluded if they (i) did not provide information for both sexes/genders separately or for either male- or female-only patients; (ii) provided mainly commentary or proposed guidelines; or (iii) did not primarily assess risk factors and preventive strategies for mental health related conditions.

## 3. Results

**Figure 1:**
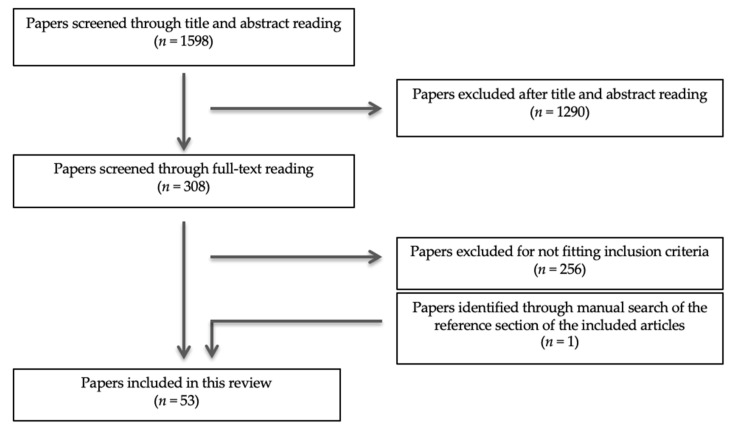
Screening process.

By using a three-step screening approach, titles, abstracts, or full texts of all records were screened against the inclusion and exclusion criteria (Figure 1). In summary, 53 articles have been included in the present review. For clarity, results have been grouped into four sections according to the specific types of mental health risk factors and potential preventive interventions as a function of gender/sex. A summary of the main findings of the papers included is presented in the tables below (Table 1, Table 2, Table 3 and Table 4). To be as informative as possible, research evidence was methodologically assessed (Supplementary Tables S1–S4). By applying a flexible approach, other articles that were deemed to cover prominent related topics have also been described throughout the present review, to provide a more comprehensive overview.

### 3.1. Individual Factors

#### 3.1.1. Childhood Adversities: Risk Factors and Long-Term Impact

Child maltreatment affects up to 80% of children worldwide [21]. Childhood abuse rates are 2–3 times higher in women compared to men [22] and victims of childhood abuse present with several negative sequelae, such as depression [23], Post-Traumatic Stress Disorder [24], suicidality [25], eating disorders [26], and drug abuse [27]. They are also at risk for re-victimization as adults and intimate partner violence [28], and their offspring are at an increased risk of being abused [29]. 

Preventing childhood abuse is challenging, partly due to the level of support needed to overcome risk factors and bolster protective factors for families and communities [30]. Most preventive interventions are based on home visits [31]. At-risk parents are usually identified among low-income families, in the context of parental young age, maternal depression and substance abuse, family stress, lack of social support, and intimate partner violence [31]. Most home-visit interventions are delivered by trained paraprofessionals or nurses [31] and start during pregnancy [32], to establish early trust between the mother and the visitor and continue up to two years after childbirth [33]. Intervention targets usually include promoting access to prenatal and pediatric care, understanding infant development, enhancing parent–infant interaction, mobilizing psychosocial support, delaying repeat pregnancy, and improving maternal life trajectory [31]. These interventions reduce child abuse rates, maternal depression, repeat pregnancy, and externalizing behaviors of children, and increase mother–infant interaction, maternal employment, and the cognitive development of children [31]. They have shown long-term protective effects on children, who display lower rates of substance use, better academic achievement, and fewer arrests and convictions [34]. Evidence on interventions other than home visitation is limited. Two studies have assessed the efficacy and cost differential of postpartum groups as compared to home visits, suggesting that groups can improve parenting knowledge [35] and maternal mental health [36] at a lower cost. However, these studies did not measure the impact upon child abuse events [31]. 

#### 3.1.2. At-Risk Children: Early Identification and Intervention

There are useful screening tools to identify and care for youth who have been exposed to violence (Table 1, upper part, and Supplementary Table S1). Once traumatic experiences are identified, Trauma-Focused Cognitive Behavioral Therapy (TFCBT) for youth should be offered. It is a structured, short-term psychotherapeutic treatment model that improves trauma-related outcomes for children/teens and their caregivers. It has been shown to have superior outcomes than other methods in different settings and with diverse populations [37,38]. No study has focused on gender-specific interventions to prevent childhood abuse. In addition, the available intervention research largely focuses on responses to traumatic experiences rather than prevention [39]. 

#### 3.1.3. Gender-Oriented Prevention of Childhood Adversities

The differential susceptibility of girls and boys to various forms of maltreatment should be addressed in dedicated preventive programs, focusing on boys and preventing harsh physical punishment and its consequences, since being a victim of physical violence in childhood increases the likeliness of becoming perpetrators of physical violence in later life [40]. Instead, preventive programs in girls should involve sexual abuse prevention, female genital mutilation prevention, neglect, and infanticide. They could reduce the incidence of adult re-victimization [31], HIV and other sexually transmitted disease infections [41], and selective abortion practices [42]. Interventions designed for preventing childhood abuse in boys and girls are likely to decrease the incidence and impact of such events and, in the long run, inequalities in the community and healthcare costs.

### 3.2. Familial Factors

#### 3.2.1. The Perinatal Period: A Sensitive Time in Life with Long-Lasting Effects

The perinatal period is characterized by several biological and psychosocial changes [43]. While most women develop confidence and satisfaction with their new roles [44], a significant proportion does experience distress. Maternal distress occurs when the infant’s demands are perceived as exceeding the available resources for coping [45], thus increasing the risk of developing mental health problems. Mental difficulties are common during the perinatal period, affecting around 16% of women during pregnancy and 20% postpartum [46]. Despite one in five women experiencing a perinatal mental health disorder, many do not receive a correct diagnosis [47]. Among women with an identified mental health problem, only 15% receive adequate treatment [48]. Perinatal mental health problems are associated with a poor mother–infant relationship, insecure attachment, and increased risk of the child developing emotional, behavioral, and cognitive problems in later life [49,50,51,52]. Since preventive interventions during early childhood are more effective than those occurring later in life [53], the perinatal period constitutes a window of opportunity for prevention. 

It is estimated that one in three women is a victim of physical, sexual, or emotional abuse by her partner [54]. Although Intimate Partner Violence (IPV) can occur at any time in life, the highest prevalence has been reported during a woman’s reproductive years [55] and more than 25% of women are pregnant when violence occurs [56]. In some cases, maternity can lead to the initiation, continuation, or increased frequency or severity of aggression [55], making IPV one of the most common health risks in the perinatal period [57]. Perinatal IPV is associated with several psychiatric disorders, especially postpartum depression [58] and child adverse outcomes [59]. Risk factors for IPV in pregnancy include abuse before pregnancy, low educational level, low socioeconomic status, being single or living apart, alcohol abuse, unintended/unwanted pregnancies, and lifetime adversity/exposure to violence [60]. Perinatal mental health problems can be minimized if women and families engage with dedicated services [61]. Key elements in promoting mental health during the perinatal period are: (i) detection of at-risk mothers; (ii) availability of effective perinatal interventions; (iii) availability of an organizational framework for the interdisciplinary work [62].

#### 3.2.2. Detection of At-Risk Mothers

The detection of at-risk mothers involves the assessment of pre-existing mental health or substance abuse disorders; screening for domestic and intimate partner violence; and evaluation of familial, social, and economic support [63]. Assessment tools to screen risk factors for perinatal mental health problems are listed in the middle part of Table 1 and Supplementary Table S1. None of them have been considered reliable for detecting antenatal mental health problems due to low positive predictive values, insufficient information regarding clinical performance, or insufficient sample size [64]. Despite limitations, they can assess the need for further intervention or referral to mental health services. Postnatal depression is common in the perinatal period, affecting up to 15% of pregnant women [46]. Approximately 40% of women will experience their first depressive episode postpartum [65] and if untreated, are more likely to experience further depressive episodes [66]. Commonly used screening tools for postnatal depression are listed in the middle part of Table 1 and Supplementary Table S1. A recent review concluded that none of these could be deemed best at detecting perinatal depression based on sensitivity and specificity. Moreover, there was no agreement on the window period for such screening tools to be administered [67]. Concerning IPV, since pregnancy involves repeated contact with healthcare providers, it offers a unique opportunity to develop trust between women and the healthcare team, increasing the possibility of disclosing abusive situations. Women may be motivated by the desire to protect their children from possible abuse by an intimate partner. Most pregnant women are accepting of enquiries regarding IPV, provided there is enough privacy and confidentiality, and the enquiries and disclosures lead to positive consequences [68]. Thus, checking any relationship stress during each antenatal and postpartum visit may be helpful. The lower part of Table 1 and Supplementary Table S1 list the most used screening tools for detecting IPV in pregnancy. Once IPV is disclosed, it is essential to determine whether the woman is in immediate danger, if substance abuse is involved, and if there is an alternate environment where the woman can ensure her safety [69]. Protocols for immediate access or referral to support are most successful in increasing IPV identification [70]. In no case should women be blamed or pressed to leave their partner [58]. 

Nevertheless, less than half of pregnant women with mental health problems are identified in clinical settings [48]. Reasons for low detection rates include inadequate training; insufficient interventions for women with psychiatric problems in the peripartum; and barriers in accessing psychiatric services due to stigma, fear of child welfare consequences and lack of adequate childcare [48].

#### 3.2.3. Availability of Effective Perinatal Interventions

These include medication, psychological treatments, and psychosocial interventions. Nearly all studies have focused on preventing perinatal depression, the most common mental health problem in the peripartum, which has potentially severe consequences. Prophylactic medication treatments in the postpartum have been tested in some small trials [71,72,73,74,75,76,77,78,79,80,81,82], whose results are summarized in the upper part of Table 2 and Supplementary Table S2. There is no specific approach recommended for preventing postpartum depression in clinical practice. Women with perinatal mental health problems indicate a preference for health promotion and psychological interventions over medications [83]. Preventive psychological treatments for perinatal depression have focused on interpersonal therapy [84,85], cognitive-behavioral therapy (CBT) [86,87] and midwife-led psychological debriefing [88,89,90,91]. Trial results are summarized in the middle part of Table 2 and Supplementary Table S2. Among psychosocial interventions for preventing postpartum depression, we found examples of trials on antenatal and postnatal classes [92,93,94,95], intrapartum support [96,97,98,99], and supportive interactions strategies based on extensive nursing home visits or additional support provided by trained postpartum workers [100,101,102,103]. Trial results are summarized in the lower part of Table 2 and Supplementary Table S2. Despite home visits and home-based psychological interventions (interpersonal therapy, CBT, and counseling) appearing to be promising for the treatment of postpartum depression [104], there is insufficient evidence to recommend them for preventing postpartum depression.

#### 3.2.4. Availability of an Organizational Framework for Interdisciplinary Interventions in Perinatal Health

Continuity of care has been associated with women’s satisfaction. Three trials have compared midwife-managed care with shared care (care divided among midwives, hospital physicians and general practitioners) in preventing postnatal depression. Out of convenience, midwives have been identified as case managers [48]. In a large trial, women in the midwife-managed group showed lower depressive levels compared to women in the shared care group [105], whereas the other two trials showed no differences in depressive levels between experimental and control groups [106,107]. While continuous midwifery care did not prevent postpartum depression, it appeared to be highly successful at engaging women in treatment [107]. Antenatal classes, early postpartum appointments, and educational strategies have been suggested as a good opportunity for delivering preventive interventions for postpartum depression in some small trials [108,109,110,111,112,113], whose results are summarized in Table 3 and Supplementary Table S3. To date, there is insufficient evidence to recommend any intervention for preventing postpartum depression. 

### 3.3. Social Factors

#### 3.3.1. Education

Formal education offers benefits for mental health and may be protective, specifically against depression [114]. Higher education may be protective against mental health problems possibly because it can lead to more fulfilling careers and higher socioeconomic positions [115]. It may also sustain healthier lifestyle behaviors and provide better access to healthcare [115]. Higher education may increase the ability to cope with stress. Moreover, it has been associated with better-perceived quality of life, albeit only in women, whereas low levels of education are associated with higher use of health services in women compared with men, as a result of increased mental health issues [116]. Three studies have shown that the protective effects of education on depressive symptoms are larger for women compared with men [117,118,119], possibly due to the greater effect of education-related work creativity and sense of control on depressive symptoms in women [117]. These results have not been replicated by recent studies [120]. It is known that school difficulties may be higher in those experiencing psychological distress [121] and, in some cases, they may be a consequence of a mental disorder [122]. School difficulties in both genders and academic failure in men are associated with an increased risk for suicide before the age of 35 [122]. Thus, schools and universities may be good places to deliver mental health prevention interventions. Even though a few mental health preventive interventions in schools have been developed [123], no study has been made on the impact of gender on mental health preventive interventions in schools and universities. 

#### 3.3.2. Employment

Women are less occupationally active than men. In 2017, among people aged 20–64, the share of working women in Europe was 66.5% while being 78% in men [124]. Studies conducted in the ’60s and the ’70s reported worse health consequences in unemployed men compared to unemployed women. Offered explanations included: first, as masculine identity has been historically linked to having a job in Western countries, unemployment was suggested to threaten it [125]; second, women have been suggested to compensate for the negative effect of unemployment by returning to their position as housewives [126]. However, with the increasing participation of women in the labor market, these results have been overturned by more recent studies indicating that unemployment has a stronger association with negative health outcomes in women than men [127,128,129]. Additionally, women tend to report lower health status, more somatic and psychological symptoms [130], and higher levels of smoking and alcohol consumption during recession periods compared with men [129]. Women with low socioeconomic status appear to be particularly hit by the consequences of recession [131], possibly because of the detrimental effect of financial strain and social isolation [132]. 

An important issue for employed women is discrimination. It is defined as being treated unfairly in any field of public life based on one’s personal characteristics, such as race, gender, or religion [133]. Perceived discrimination is strongly associated with poor indicators of both physical [134] and mental health, particularly with anxiety [135], depression [136] and PTSD [137]. Gender discrimination in the workplace includes harassment, unequal pay, and the implementation of rules that put one gender at a disadvantage [12]. Statistics from Europe show that women earn on average 20% less than men [138]. More than 50% of employed women report being victims of sexual harassment in the workplace [139]. Evidence shows gender-biased hiring preferences with men being favored over women, even though qualifications and experience are identical [140]. Gender differences in the labor market are posited to be associated with differences in mental health problems reported by men and women [141]. Few studies have focused on workplace-related gender discrimination [136]. One large cohort study [142] has focused on the effect of age discrimination on mental health in the workplace and found that perceived age discrimination is a significant predictor of women’s depressive symptoms and life satisfaction over the life course. 

Male-dominated industries (i.e., industries in which more than 70 percent of workers are men, such as agriculture, construction, mining, and utilities) [143] have higher than average rates of anxiety and mood disorders. Men are more reticent to access professional mental health services and seek help for psychological problems [144]. This may be due to “traditional masculine behavior” [145], the perceived stigma around mental health [146], and employment status [147]. Therefore, workers in male-dominated industries are at higher risk of mental health disorders but less likely to access treatment [148]. Interventions delivered in settings with a high male prevalence, including the workplace, hold promise for promoting mental health among men [149]. Key elements to promote mental health among workers in male-dominated industries include distribution of information to workers about mental health issues, provision of additional social support, access to treatment and advice for workers, education for managers about mental health in the workplace, addressing excessive workloads, providing relief periods from heavy workloads, team environment development, and increased job demand, job variety and control [148,150]. The economic climate, labor market conditions, employment policies, and job security can contribute to workers’ psychological wellbeing [151]. Interventions that target the whole workplace, utilize team-based approaches, and use multiple strategies are the most effective [152]. It is difficult to compare results between different studies promoting mental health among men in workplaces, due to the large variety of measures used and psychological outcomes examined [153]. The most investigated outcome measure was stress reduction [154,155,156,157]. Studies show that mental health promotion initiatives are more effective in female workers [156,157], possibly because men are less likely to participate in planned workshops.

#### 3.3.3. Relationships

Some early studies show that married women exhibit higher rates of mental disorders compared with men, while others report that the mental health benefits of being married extend equally to men and women [158]. Marriage may confer benefits to mental health because it provides an intimate, emotionally fulfilling relationship that satisfies an individuals’ needs for social integration and support [159]. Spouses also monitor one another, encouraging healthy behaviors that promote emotional wellbeing [160]. Marriage can also create an important sense of identity and purpose for many people, increasing mental wellbeing [161]. Entry into marriage is associated with lower levels of distress, whereas a transition out of marriage increases psychological distress in both sexes [162]. Women report a greater increase in psychological distress than men when their marriages break down [163]. Increased vulnerability to distress may also occur for women who become widowed or re-married [162]. There is evidence that the costs of loss and separation or divorce fall more heavily on women than men [164]. Given the potential benefits of marriage, some countries have developed programs to promote healthy marriages via relationship education as a strategy to improve public health. Key elements are couple and marriage education, and support for adults; relationships and marriage education for high school students; and fatherhood programs with co-parenting or marriage components (Table 4 and Supplementary Table S4). Programs include capacity-building activities and public education, community awareness, and outreach components of service programs, and they have shown to be effective in increasing the longevity of marriages [165].

### 3.4. Healthcare Factors

Gender differences in mental health service use are known, with men being less likely to seek services for mental health compared to women [166]. Proposed solutions to increase access to mental health services include integrating behavioral health services into primary or community-based care, augmenting the workforce through task-shifting (e.g., utilizing community health workers or peer navigators to provide some services), imparting training and supervision to novel providers via the Internet, or delivering services to people where they live (e.g., via minute clinics, medical vans, or tele-mental health services) [167]. None of these solutions has involved a gendered perspective.

## 4. Discussion

This is the first review attempting at unifying evidence on gender- and sex-based mental health risk factors, early identification of at-risk individuals, and prevention strategies. Inter-individual differences in mental health are known and there is an agreement that mental health risk trajectories may sensibly differ as a function of gender- (e.g., social role) and sex-related (e.g., hormonal) effects [12]. There is a limited availability of mental health prevention strategies that are specifically designed to consider gender- and sex-based differences and needs. This is a result of the paucity of high-quality trials and longitudinal studies informing on the best strategies to sustain mental health and prevent adverse outcomes, based on the psychosocial and neurobiological differences between men and women.

Early childhood represents one of the more important and most investigated areas for mental health prevention, which stems from the evidence that the neurodevelopmental period offers the greatest chances to positively shape an individual’s future at multiple levels [18]. Conversely, in the presence of genetic and environmental affronts during childhood, several non-specific psychosocial disturbances may occur, compromising the child’s wellbeing and leading to the manifestation of a frank mental condition between early adolescence and early adult years [168]. Mental disorders and related conditions account for 45% of the global burden of disease across the 0–25 year old age span [168]. Results from an international survey conducted among 51,945 adults indicate that eradicating childhood adversities would reduce the occurrence of any mental disorder by one-third [169]. Consistent, mental health preservation strategies during childhood are mainly focused on childhood abuse, however, they do not consider gender differences in childhood abuse rates and effects. 

Most sex-specific preventive strategies are focused on pregnancy and post-partum as well as related familial issues, and mainly address depressive symptoms. About twice as many women as men experience depression [170]. Risk factors increasing women’s risk of depression include psychobiological changes that occur during puberty, premenstrual problems, pregnancy, perimenopause, menopause, and life circumstances and cultural stressors [171]. Hormonal fluxes occurring in the perinatal period, along with the psychosocial changes surrounding pregnancy, are windows to pay attention to when implementing sex-based preventive interventions. In a wider perspective, research evidence converges on the importance of promoting a positive social climate within the family to sustain individuals’ wellbeing [172]. 

Regarding social factors, employment is an area contributing to shaping life and identity. Despite evidence of gender-related differences, inequalities, and discrimination in the workplace [173], there are limited gender-specific mental health prevention programs for working and employment conditions. Some strategies have been developed for men in male-dominated industries, focusing on stress reduction. Concerning relationships, marriage is not unequivocally associated with positive or negative effects for mental health. Possibly, marital relationships confer both resources and strains, with effects in terms of the couple’s mental health that depend on the balance between these factors [158]. It is much clearer that marriage disruption may have detrimental consequences, especially for women’s mental health and coping strategies. There are some gendered mental health preventive strategies to support healthy marriages. It is known that men are more reluctant than women to access mental health services, however, limited efforts have been put in developing strategies that guarantee equal access to care. It is worth mentioning that people with non-conforming gender may suffer from psychobiological distress [174,175,176] possibly due to barriers to healthcare access [13]. It is imperative to try and personalize access to care depending on the individual’s intrinsic needs.

**Figure 2:**
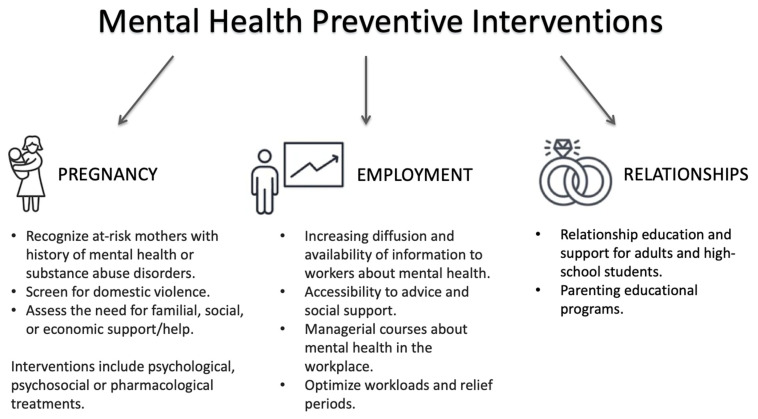
Summary of available mental health preventive interventions.

The present review offers a reappraisal of evidence on the role of gender and biological sex in mental health prevention (graphically synthesized in Figure 2). It must be viewed while considering some limitations. Research in the field is still too limited, with varied and heterogeneous findings. Performing such a review required a suitably inclusive and flexible approach to provide the most comprehensive evidence on gender- and sex-oriented mental health prevention strategies. However, studies’ methodological heterogeneity limited our ability to systematically review the existing scientific evidence, carrying a high risk of bias and making it difficult to draw comparative conclusions and offer indications for prevention as a function of gender or sex. In some cases, evidence was particularly fragmented. In the absence of longer-term investigations, the effectiveness of gender-related specific prevention strategies remains unclear.

## 5. Conclusions

Prevention and screening strategies can have a remarkable impact on individuals’ mental health and general wellbeing. Their implementation should not fail to consider that risk factors for mental health-related conditions display gender/sex differences. Such gender/sex differences have rarely been considered and included in mental health preventive programs. Most evidence comes from studies in adult populations (e.g., postpartum depression), while research on the early phases of life is lacking. More research is needed on how gender differences interact with biological and physiological characteristics in influencing mental health needs, the risk of developing a mental condition, mental health service access and utilization, and responses to pharmacological and non-pharmacological treatments.

## Figures and Tables

**Table 1:** Screening tools for prevention and early intervention in mental health.

Tool	No. of Items	Characteristics
Childhood abuse		
Trauma Symptom Checklist for Children/Trauma Symptom Checklist for Young Children (TSCC/TSCYC)	20	The clinical scales include PTS-Intrusion, PTS-Avoidance, PTS-Arousal, Sexual Concerns, Anxiety, Depression, Dissociation, and Anger/Aggression.
UCLA PTSD Reaction Index (UCLA PTSD-RI)	12	It includes parent-report and self-report versions. It asks individuals to identify the current most impairing event and asks questions about the child’s reactions during or directly after exposure to that event. Finally, it assesses PTSD symptom frequency on a 5-point Likert scale within the past month.
Child PTSD Symptom Scale (CPSS)	26	Respondents indicate how often they experienced each symptom in the past month on a 4-point Likert scale from 0 (not at all) to 3 (5 or more times a week).
Perinatal mental health problems		
Antenatal Psychosocial Health Assessment (ALPHA)	35	It identifies antenatal psychosocial risk factors that would lead to poor postnatal psychosocial outcomes. Questions are scored using a three-point tick-box system of ‘low’, ‘some’ and ‘high’.
Antenatal Risk Questionnaire (ANRQ)	12	It assesses the following psychosocial risk domains: emotional support from subject’smother in childhood, past history of depressed mood or mental illness and treatment received, perceived level of support available following the birth of the baby, partner emotional support, life stresses in the previous 12 months, personality style (anxious or perfectionistic traits) and history of abuse (emotional, physical and sexual).
Australian Routine Psychosocial Assessment (ARPA)	12	The tool assesses support, stressors, personality, mental health, childhood abuse, family violence and current mood.
Camberwell Assessment of Need—Mothers (CAN-M)	26	It covers the domains of accommodation, food, looking after the home, self-care, daytime activities, general physical health, pregnancy care, sleep, psychotic symptoms, psychological distress, information, safety to self, safety to child and others, substance misuse, company, intimate relationships, sexual health, violence and abuse, practical demands of childcare, emotional demands of childcare, basic education, telephone, transport, budgeting, benefits, language, culture and religion. Domains are assessed on a five-point Likert scale of importance (ranging from ‘not at all’ to ‘essential’).
Contextual Assessment of Maternity Experience (CAME)	3	It explores recent life adversity or stressors, the quality of social support and key relationships including partner relationship, and maternal feelings towards pregnancy, motherhood and the baby.
Pregnancy risk questionnaire (PRQ)	18	It assesses mother’s attitude to her pregnancy, mother’s experience of parenting in childhood, history of physical or sexual abuse, history of depression, the impact of depression on psychosocial function, whether treatment was sought or recommended, presence of emotional support from partner and mother, presence of other supports, presence of stressors during pregnancy, trait anxiety, obsessional traits and self-esteem. A five-point Likert scale is used, from 1 ‘not at all’ to 5 ‘very much’.
Postnatal depression		
Edinburgh Postnatal Depression Scale (EPDS)	10	It is the most widely tested screening tool for postnatal depression, although its sensitivity varies from 22% to 96%. Possible scores range from 0 to 30, with 11 and 13 being the most commonly used cut-offs to detect “probable” depression. It limits questions to feelings of sadness or anxiety, without screening for physical symptoms. Its reference period is narrow since it allows patients to report symptoms felt during the week before the assessment.
Postpartum Depression Screening Scale (PDSS)	35	It assesses Sleeping/Eating Disturbances, Anxiety/Insecurity, Emotional Lability, Cognitive Impairment, Loss of Self, Guilt/Shame, and Contemplating Harming Oneself. On completing the scale, a mother is asked to select a label from (1) to (5) to reflect her degree of disagreement or agreement, where (1) means strongly disagree and (5) means strongly agree.
Beck’s Depression Inventory-II (BDI-II)	21	It measures the severity of depression with four response options ranging from 0 to 3 for each item, with a total maximum score for all items being 63. A score of 0–13 is considered minimal, 14–19 mild, 20–28 moderate, and 29–63 is considered severe depression.
General Health Questionnaire-12 (GHQ-12)	12	It has four response options and an overall rating from 0 to 12 used to assess mental health and psychological adjustment.
Center for Epidemiological Studies Depression Scale (CES-D)	20	It is a Likert-format screening tool that asks respondents how often they experienced a particular symptom in the past week, where 0 represents “rarely or none of the time” and 3 represents “most or all of the time” (range 0–60).
Patient Health Questionnaire (PHQ)	9	It assesses the experiencing of depressive symptoms over the last 14 days. Scores on the PHQ-9 range from 0 to 27 and are calculated by assigning scores of 0, 1, 2 or 3 to response categories of ‘not at all’, ‘several days’, ‘more than half the days’ or ‘nearly every day’, respectively and then summing up the scores.
Intimate partner violence		
RADAR	5	It is an acronym-mnemonic that helps summarize key action steps that physicians should take in recognizing and treating patients affected by IPV. The tool includes (1) Routinely screening adult patients, (2) Asking direct questions, (3) Documenting your findings, (4) Assessing patient safety, and (5) Reviewing options and referrals.
HIITS	5	The tool asks a patient the following questions: How often does your partner physically hurt you, insult or talk down to you, threaten you with harm, and scream or curse at you? Each category is graded on a scale of 1 (never) to 5 (frequently) and a sum of all the categories is generated. A total score of 10 or above is suggestive of IPV.
Abuse Assessment Screen (AAS)	5	It involves the following open-ended questions: 1. Have you ever been emotionally or physically abused by your partner or someone important to you? 2. Since I saw you last have you been hit, slapped, kicked, or otherwise physically hurt by someone? If YES, by whom? Number of times? Nature of injury? 3. Since you have been pregnant, have you been hit, slapped, kicked, or otherwise physically hurt by someone? If YES, by whom? Number of times? Nature of injury? 4. Within the past year has anyone made you do something sexual that you did not want to do? If YES, then who? 5. Are you afraid of your partner or anyone else?

**Table 2:** Mental health care in the perinatal period.

Intervention	Author(s)	Main Findings
Biological		
Prophylactic medication in the postpartum with Nortriptyline	Wisner et al., 1994 Wisner et al., 2001	In one study, prophylactic Nortryptiline appeared to be effective in reducing postpartum depression relapse at 12 weeks postpartum (Wisner et al., 1994), whereas the other study found no difference in depressive levels at 20 weeks postpartum between women taking the antidepressant versus controls (Wisner et al., 2001).
Prophylactic effect of estrogen and progesterone therapy in preventing postpartum depression	Sichel et al., 1995 Dalton et al., 1994 Dalton et al., 1976 Lawrie et al., 1998	Results were promising for prophylactic estrogen therapy (Sichel et al., 1995) but highly inconsistent for prophylactic progesterone therapy, with two small studies showing a reduction in the postpartum depression recurrence rate (Dalton et al., 1994–1976) and another larger trial showing an increased risk of developing depressing symptoms in women taking part in progesterone therapy compared to controls (Lawrie et al., 1998).
Thyroid antibodies in the postpartum	Harris et al., 2002	A small trial failed to show an effect in the occurrence of depression in thyroid-antibody-positive women taking thyroxine postpartum compared to thyroid-antibody-positive women taking a placebo.
Docosahexanoic Acid (DHA) in postpartum	Llorente et al., 2003	A small trial did not show a significant effect on postpartum depression rates.
Calcium supplementation	Harrison-Hohner et al., 2001	Promising effect in preventing postpartum depression in a small trial, since calcium metabolism is influenced by fluctuations in gonadal hormones that are exacerbated in the postpartum period.
Psychological		
Interpersonal therapy	Zlotnick et al., 2001 Gorman et al., 2001	Interpersonal therapy appeared to be effective in preventing depression compared to controls at four weeks postpartum, but this prophylactic effect was not maintained at 24 weeks postpartum (Gorman et al., 2001).
Cognitive-behavioral therapy	Chabrol et al., 2002	One study showed that CBT is efficacious and well-accepted for post-partum depression
Midwife-led psychological debriefing	Lavender et al., 1998 Small et al., 2000 Priest et al., 2003 Gordon and Gordon, 1960 Elliott et al., 2000	Midwife-led debriefing appeared to be effective in lowering anxiety and depression scores in the postnatal period (Lavender et al., 1998). In one study, women in the psychological debriefing group presented with less depressive symptoms at 3 weeks postpartum compared to controls (Small et al., 2000), in another study women in the experimental group showed higher levels of depressive symptoms at 24 weeks postpartum compared to controls (Priest et al., 2003), and in the remaining two studies no difference in depressive levels was found between treated woman and controls (Gordon and Gordon, 1960; Elliott et al., 2000).
Psychosocial		
Antenatal classes	Stamp et al., 1995 Brugha et al., 2000 Buist et al., 1999	Effective in preventing postpartum depression only in one trial (Stamp et al., 1995), whereas in two studies no differences were found in depressive levels between experimental and control groups (Brugha et al., 2000; Buist et al., 1999).
Intrapartum support	Wolman et al., 1993 Nikodem et al., 1998 Gordon et al., 1999 Hodnett et al., 2002	Effective in preventing postpartum depression at 6 weeks but not at 1 year postpartum (Wolman et al., 1993; Nikodem et al., 1998), the positive effect at 6 weeks postpartum was not replicated in other studies (Gordon et al., 1999; Hodnett et al., 2002)
Interaction strategies	Armstrong et al., 1999 Armstrong et al., 2000 Morrell et al., 2000 Reid et al., 2002	These include extensive nursing home visits (Armstrong et al., 1999–2000) or additional support provided by trained postpartum workers (Morrell et al., 2000; Reid et al., 2002). They showed a reduction in depressive levels at 6 weeks postpartum compared to controls, but these results were not maintained at follow-up assessments.

**Table 3:** Organizational framework for interdisciplinary interventions in perinatal health.

Intervention	Author(s)	Main Findings
Antenatal classes	Webster et al., 2003	A randomized controlled trial showed no differences in depression levels between experimental and control groups at 16 weeks postpartum.
Early postpartum appointments	Serwint et al., 1991 Gunn et al., 1998	Appointments were delivered 2–6 weeks postpartum in order to prevent postpartum depression and appeared to be only slightly effective in reducing depressive levels compared to controls.
Educational strategies	Okano et al., 1998 Heh et al., 2003 Hayes et al., 2001	In two trials they were successful in decreasing the severity of postpartum depression and the time between onset of depressive symptoms and seeking professional help (Okano et al., 1998; Heh et al., 2003). However, a larger trial failed to replicate the result (Hayes et al., 2001).

**Table 4:** Key elements of healthy marriage promotion.

Intervention	Description
Couples and marriage education programs	Changing attitudes and dispeling myths about marriage and teach relationship skills, especially related to communication and conflict resolution for adults at various life stages: single, dating, engaged, newly married, marriage in crisis, and those who are remarried.
Relationship education for students	Teaching middle and high school students about skills for building successful relationships and marriages.
Fatherhood programs	Promoting the importance of fatherhood and helping fathers to become more involved with their children. They encompass job training and placement, child support payment assistance, peer support groups, parenting classes, legal assistance, and individual counseling.

## Data Availability

Not applicable.

## Supplementary Materials

The following supporting information can be downloaded at: https://www.mdpi.com/article/10.3390/ijerph19031493/s1, Table S1: Summary of studies discussing available screening tools and strategies for mental health prevention and early intervention; Table S2: Summary of studies investigating postpartum depression preventive interventions in the perinatal period; Table S3: Summary of studies investigating postpartum depression preventive interventions using antenatal classes, early postpartum appointments and educational strategies; Table S4: Summary of studies investigating the impact of marriage on mental health and key elements of healthy marriage promotion.
