# Supplementary material for: Gender-Oriented Mental Health Prevention: A Reappraisal
Source: Int J Environ Res Public Health. 2022 Jan 28;19(3):1493. doi: 10.3390/ijerph19031493 (PMC8835536; doi:10.3390/ijerph19031493)
Supplement: Supplementary file 1 [file ijerph-19-01493-s001.zip › ijerph-1539234-supplementary.pdf]

Supplementary Table S1. Summary of studies discussing available screening tools and strategies for mental health prevention and early intervention.

Study	Study Design	Aim of the Study	Population	N	Age (Years)	Gender	Results
Levey et al., 2017 [31]	√ Systematic review of RCTs	√ Provide an overview of existing literature on the effectiveness of interventions aimed at reducing child abuse in high-risk families	√ Pregnant women/new mothers at high risk of abusing their child(ren)	√ 8 US-UK RCTs with different sample sizes (93–1173)	X	√ Female	Women with history of abuse may benefit from distinct interventions and need specific types of support to help them access interventions; for now, only home visitation has significant evidence base for decreasing childhood abuse
Murray et al., 2015 [37]	√ Randomized controlled trial	√ Assess the effectiveness of TF-CBT among children	√ Zambian children with ≥1 traumatic event and trauma-related manifestations (score of ≥1 on UCLA PTSD-RI): 131 in INT + 126 in CTL	√ 257	√ Mean (SD): 1. INT: 14.02 (2.77); 2. CTL: 13.29 (2.99)	√ Female/Male	TF-CBT significantly reduced stress and trauma-related symptoms
Cohen et al., 2007 [38]	√ Pilot randomized controlled trial	√ Investigate the effectiveness of Sertraline + TF-CBT to improve PTSD in children with history of sexual abuse through the use of different instruments (CPSS, K-SADS-PL, CGAS, MFQ, SCARED, CBCL, BDI, Parent's Emotional Reaction Questionnaire, SEF-CA)	√ 24 female children and adolescents and their caregiver randomly assigned to receive TF-CBT + Sertraline or TF-CBT + placebo for 12 weeks: 12 in INT + 12 in CTL	√ 24	√ 10–17 yo. No. (%)<br>- 10–11: 5 (22.7)<br>- 12–14: 10 (45.5)<br>- 15–17: 7 (31.8)	√ Female	No significant differences between INT and CTL
MacMillan et al., 2007 [40]	√ Literature review	√ Describe the development of a proposed national child maltreatment research strategy	X	X	X	X	Childhood abuse prevention programs for boys should be more focused on preventing harsh physical punishment and its consequences
Wagman et al., 2015 [41]	√ Community-based cohort study	√ Investigate if a combination of IPV prevention and HIV services provision	√ 11448 females and males: 6702 F + 4746 M	√ 11448	√ 15–49	√ Female/Male	Exposure to SHARE significantly reduced IPV and HIV rates

		may help reduce IPV and HIV incidence	5337 in INT + 6111 in CTL				
Choi et al., 2020 [63]	√ Commentary	√ Present recommendations to promote maternal and infant mental health in the perinatal period	X	X	X	X	Promotion of mental health in the perinatal period involves encouraging social support, infant attachment, and assessment of familial, social, and economic support
Johnson et al., 2012 [64]	√ Systematic review (24 papers)	√ Analyze existing tools (ALPHA, ANRQ, ARPA, CAN-M, CAME, PRQ) to assess the perinatal mental health risk	X	X	X	X	These tools can be used in clinical practice to assess the need for further intervention or access to mental health services; however, their effectiveness in adequately detecting antenatal mental health problems is still debated
Woolhouse et al., 2015 [65]	√ Prospective pregnancy cohort study of nulliparous women	√ Describe maternal depression risk factors and prevalence from pregnancy to 4 years PP	√ 1507 Australian nulliparous women registered to give birth at 6 public Hospitals	√ 1507	√ ≥18 years	√ Female	Maternal depression is common 4 years PP, thus there is the need for surveillance of mothers mental health beyond the perinatal period
Josefsson et al., 2007 [66]	√ Longitudinal study	√ Assess PPD prevalence in women with previous PP depressive manifestations	√ 675 women with PPD on the EPDS: 221 in INT + 454 in CTL	√ 675	√ Mean (SD): 1. INT: 33.5 (5.0); 2. CTL: 33.3 (4.5)	√ Female	PPD represents a risk for further episodes of depression later in life, thus early recognition and intervention is needed to prevent/reduce long-term consequences
Ukatu et al., 2018 [67]	√ Literature review (12 review articles: 2	√ Investigate PPD screening tools accuracy (PDSS, PRQ, BDI-II, EPDS, GHQ-12,	X	X	X	X	None of the analyzed tools could be deemed best at detecting PPD based

	retrospective studies + 10 systematic reviews)	CES-D, patient Health Questionnaire)					on sensitivity and specificity
Stöckl et al., 2013 [68]	√ Mixed method study	√ Investigate if pregnant women are accepting of enquiry on IPV, using a self-administered questionnaire and in-depth interviews with women having a history of violence during pregnancy; a modified version of the AAS was used to evaluate psychological and physical abuse, before and during pregnancy	√ 401 pregnant German women	√ 401	√ Mean (SD) [range]: 33 (5.4) [18–50]	√ Female	Women are highly supportive of routine or case-based inquiry for IPV in antenatal care, provided there is enough privacy and confidentiality. Thus, checking any relationship stress during each antenatal and postpartum visit may be helpful
Deshpande et al., 2013 [69]	√ Review	√ Investigate valid assessment and screening tools (RADAR tool, AAS tool and HITS tool) to detect IPV in pregnant women	X	X	X	X	Since IPV is associated with multiple adverse health consequences, pregnant women should be routinely screened and structured screening tools may detect it better than a standard patient interview
Renker et al., 2006 [70]	√ Correlational design	√ Address the need for evidence-based violence screening practices, by assessing women's views on prenatal violence screening. AAS and Women's Experiences With Battering Scale were used to inquire about emotional and physical/sexual abuse	√ 519 African-American and white women who have delivered infants in stable conditions in the previous 72 h	√ 519	√ ≥18	√ Female	The majority of pregnant women are accepting of enquiry on IPV

RCT, Randomized Controlled Trial; TF-CBT, Trauma-Focused Cognitive Behavioral Therapy; UCLA PTSD-RI, UCLA Post-Traumatic Disorder Reaction Index; INT, Intervention group; CTL, Control group; CPSS, Child PTSD Symptom Scale; K-SADS-PL, Schedule for Affective Disorders and Schizophrenia for School-Age Children-Present and Lifetime version; CGAS, Children's Global Assessment Scale; MFQ, Mood and Feeling Questionnaire; SCARED, **S**creen for Children's Anxiety Related Emotional Disorders; CBCL, Child Behavior Checklist**;** BDI, Beck Depression Inventory; SEF-CA, Side Effects Form for Children and Adolescents; IPV, Intimate Partner Violence; SHARE, Safe Homes and Respect for Everyone; ALPHA, Antenatal Psychosocial Health Assessment; ANRQ, Antenatal Risk Questionnaire; ARPA, Australian Routine Psychosocial Assessment; CAN-M, Camberwell Assessment of Need—Mothers; CAME, Contextual Assessment of Maternity Experience; PRQ, Pregnancy Risk Questionnaire**;** PP, Postpartum; PPD, Postpartum depression; EPDS, Edinburgh Postnatal Depression Scale; PDSS, Depression Screening Scale; GHQ-12, General Health Questionnaire-12; CES-D, Center for Epidemiological Studies Depression Scale; AAS, Abuse Assessment Screen.

Supplementary Table S2. Summary of studies investigating postpartum depression preventive interventions in the perinatal period

Study	Study Design	Aim of the Study	Population	N	Age (Years)	Gender	Results
Wisner et al., 1994 [71]	√ Open clinical trial	√ Test the efficacy of antidepressants in the PP period to prevent recurrent PPMD	√ 23 pregnant women with ≥1 previous PP episode fitting criteria for non-bipolar major depression	√ 23	X	√ Female	Prophylactic Nortryptiline appeared to be effective in reducing relapse of PPD at 12 weeks PP
Wisner et al., 2001 [72]	√ Randomized double-blind clinical trial	√ Test the efficacy of nortriptyline in preventing recurrent PPMD	√ 51 non-depressed women with ≥1 episode of PPMD recruited during pregnancy	√ 51	√ 21–45	√ Female	Nortriptyline did not show any difference in preventing recurrent PPMD compared to placebo
Sichel et al., 1995 [73]	√ Open, pilot trial	√ Test the efficacy of estrogen prophylaxis in the treatment of recurrent PP affective disorders	√ 11 pregnant women, 7 with previous psychosis and 4 with previous MD	√ 11	√ 23–34	√ Female	Women taking prophylactic estrogens showed lower rates of relapses of PP affective disorder
Dalton et al., 1994 [74]	X	√ Test the efficacy of prophylactic progesterone therapy in preventing PPD	X	X	X	X	Reduction in the PPD recurrence rate
Dalton et al., 1976 [75]	X	√ Evaluate the effects of progesterone on PPMD compared to those of progestogens	X	X	X	X	Progesterone is identified as the drug of choice for PPMD, whereas progestogens are the choice for contraception

Lawrie et al., 1998 [76]	√ Double-blind randomized placebo-controlled trial	√ Test the efficacy of postnatal administration of norethisterone enanthate on PPD	√ 180 postnatal women using a non-hormonal contraceptive method: 90 INT + 90 CTL	√ 180	√ Mean (SD): 1. INT: 32.6 (5.9); 2. CTL: 32.3 (5.0)	√ Female	Increased risk of PPD symptoms in women taking progesterone compared to CTL
Harris et al., 2002 [77]	√ Double-blind randomized placebo-controlled trial	√ Determine if taking thyroxine in the PP period has an effect on the occurrence of depression	√ 342 thyroid-Ab positive women	√ 342 (out of 446 randomized)	√ Mean: 1. INT: 29.5 [20–42]; 2. CTL: 29.4 [19–44]	√ Female	PP thyroxine supplementation did not have an effect on PPD rates compared to placebo
Llorente et al., 2003 [79]	√ Prospective longitudinal study	√ Test the effect of DHA supplementation on PPD	√ 138 pregnant women with no history of chronic MDs and planning to breastfeed their infants	√ 138	√ 18–42 Mean (SD): 1. INT: 31.2 (4.28); 2. CTL: 31.7 (4.86)	√ Female	DHA showed no significant effect on PPD rates
Harrison-Hohner et al., 2001 [81]	√ Randomized, double-blind, placebo-controlled trial	√ Determine if prenatal calcium supplementation has an effect on PPD	√ 779 subjects in two CPEP sites: 497 from Portland, OR and 282 from Albuquerque, NM	√ 779	√ Mean (SD): 1. Portland: (a) INT: 22.2 (4.6); (b) CTL: 21.2 (4.1). 2. Albuquerque: (a) INT: 22.3 (4.7); (b) CTL: 23.1 (5.2)	√ Female	Prenatal calcium supplementation may represent an option for reducing the risk of PPD
Zlotnick et al., 2001 [84]	√ Pilot randomized controlled trial	√ Assessing the efficacy of an interpersonal-therapy-oriented group intervention in decreasing PPD rates	√ 37 pregnant women at 20–32 WOG on public assistance and with ≥1 risk factor for PPD: 18 in INT + 19 in CTL	√ 37	√ Mean (SD): 23.4 (4.41) [18–38]	√ Female	Interpersonal therapy appeared to be successful in preventing PPD over a PP period of 3 months

Gorman et al., 2001 [85]	√ Randomized controlled trial	√ Assess the efficacy of interpersonal psychotherapy in preventing PPD	√ 45 US pregnant women at risk for PPD: 24 in INT + 21 in CTL	√ 45	X	√ Female	The effects of interpersonal therapy on the occurrence of PPD were not maintained at 24 weeks PP
Chabrol et al., 2002 [87]	√ Randomized controlled study	√ Evaluate the efficacy of cognitive-behavioral therapy for prevention and treatment of PPD	√ 241 French women with EPDS scores ≥ 9: 113 in INT + 128 in CTL	√ 241	√ Mean (SD): 1. INT: 30.3 (4); 2. CTL: 29.6 (5)	√ Female	Women assigned to the prevention group showed a significant decrease in the frequency of probable depression, compared to CTL
Lavender et al., 1998 [88]	√ Randomized controlled trial	√ Evaluate the efficacy of midwife-led debriefing in decreasing PP psychological morbidity	√ 114 primiparous women: 58 in INT + 56 in CTL	√ 114 (out of 120 randomized)	√ Mean (SD): 1. INT: 24.6 (4.8); 2. CTL: 23.7 (5.8)	√ Female	Women who received the intervention showed less probability of manifesting depression and anxiety over the PP period compared to CTL
Small et al., 2000 [89]	√ Randomized controlled trial	√ Evaluate the efficacy of midwife-led debriefing in reducing the occurrence of depression at 6-months PP	√ 1041 women with an operative birth: 624 who gave birth by C-section + 353 with the use of forceps + 64 by vacuum extraction	√ 1041	√ %: 1. INT: 10% <25, 64% 25–34, 27% ≥35; 2. CTL: 12% <25, 61% 25–34, 25% ≥35.	√ Female	Midwife-led psychological debriefing did not show to be effective in reducing depressive symptoms at 6 months PP
Priest et al., 2003 [90]	√ Randomized single-blind controlled trial	√ Assess the effectiveness of midwife-led critical incident stress debriefing in reducing PPD	√ 1745 women with healthy infants born at term: 875 in INT + 870 in CTL	√ 1745	√ Maternal age >18	√ Female	Women in INT showed higher levels of depressive symptoms at 24 weeks PP compared to CTL
Gordon and Gordon, 1960 [91]	√ Quasi-experimental study	√ Assess the effectiveness of antenatal classes in decreasing the occurrence of PPD	√ 161 pregnant women: 85 in INT + 76 in CTL	√ 161	X	√ Female	No significant difference in depressive symptoms between treated women and CTL
Elliott et al., 2000 [92]	√ Controlled trial	√ Evaluate the effectiveness of a psychosocial intervention in preventing PPD	√ 99 pregnant women designated as more vulnerable by LQ or CCEI: 47 in INT + 52 in CTL	√ 99	X	√ Female	Significant group differences were shown for first-time mothers favoring the INT. Surviving Parenthood groups were unsuccessful for second-time mothers

Stamp et al., 1995 [93]	√ Randomized controlled trial	√ Assess the effectiveness of antenatal and postnatal interventions to overcome the occurrence of PPD	√ 144 women identified as more vulnerable after completion of a modified antenatal screening questionnaire: 73 in INT + 71 in CTL group	√ 144	√ Mean: 1. INT, 25.56; 2. CTL, 27.54	√ Female	No significant differences between INT and CTL
Brugha et al., 2000 [94]	√ Randomized controlled trial	√ Evaluate the effectiveness of antenatal psychosocial support in the prevention of PPD	√ 209 pregnant women identified to be at high risk for post-natal depression by a modified GHQ-D: 103 in INT + 106 in CTL	√ 209	√ 16–38	√ Female	No significant differences between INT and CTL
Buist et al., 1999 [95]	√ Pilot randomized controlled trial	√ Pilot an interventional program focused on parenting and coping strategies to prevent PPD	√ 44 Australian primiparous women identified to be at high-risk for postnatal depression based on risk factors from a review of search on PND (scores ≥8): 23 in INT + 21 in CTL	√ 44	√ Mean: 1. INT, 28.2; 2. CTL, 28.3	√ Female	No significant differences between INT and CTL
Wolman et al., 1993 [96]	√ Randomized controlled study	√ Assess the effectiveness of intrapartum support in preventing PPD	√ 189 nulliparous women labouring alone: 92 in INT + 97 in CTL	√ 189	√ Mean (SEM): 1. INT, 20.5 (0.36); 2. CTL, 20.3 (0.28)	√ Female	Intrapartum support is effective in preventing depression at 6 weeks but not at 1 year PP
Nikodem et al., 1998 [97]	√ Randomized controlled trial	√ Evaluate if supportive companionship by a doula has long-term positive psychosocial effect on the mother	√ 262 low-risk nulliparous women with cervical dilatation <6 cm and with no supportive partner with them: 126 in INT + 136 in CTL	√ 262	√ Mean: 21 for both INT and CTL	√ Female	Significant differences between INT and CTL were found at 6 weeks but not at 52 weeks PP
Gordon et al., 1999 [98]	√ Randomized controlled trial	√ Assess the effects of doula-assisted labour on PPD	√ 314 nulliparous women delivering in an HMOH: 149 with doulas + 165 with usual care	√ 314	√ %: 1. INT: 79.9% aged 18–34 and 22.1% ≥35; 2. CTL: 79.4%	√ Female	No significant differences between INT and CTL

					aged 18–34 and 20.6% ≥35.		
Hodnett et al., 2002 [99]	√ Randomized controlled trial	√ Evaluate the effectiveness of nurses providing intrapartum support in preventing PPD	√ 6915 US and Canadian women ≥34 WOG, in established labour: 3454 assigned to receive continuous labour support + 3461 receiving usual care	√ 6915	√ Mean (SD): 1. Continuous labour support group: 29.4 (5.5 [15.2–45.8]); 2. Usual care support group: 29.5 (5.7) [15.2–47.7]	√ Female	No significant differences between the two groups
Armstrong et al., 1999 [101]	√ Randomized double-blind controlled trial	√ Evaluate the effectiveness of nursing home visits targeting families where the child was at high risk for poor health and developmental outcomes	√ 181 Australian families: 90 randomly assigned to receive a program of nurse home visiting + 91 receiving usual community child health services	√ 181	√ Maternal age <18	√ Female/Male	Significant group differences were found at 6 weeks PP favoring INT, but the results were not maintained at follow-up assessments
Armstrong et al., 2000 [100]	√ Randomized controlled trial	√ Assess the effectiveness of early home visits on the quality of maternal-child attachment, maternal mood and child health	√ 181 families in the postnatal period: 90 randomly assigned to INT + 91 assigned to CTL	√ 181	√ Maternal age <18	√ Female/Male	Significant group differences were found at 6 weeks PP favoring INT, but the results were not maintained at follow-up assessments
Morrell et al., 2000 [102]	√ Randomized controlled trial	√ Assess whether women's general health could benefit from additional postnatal support by trained workers	√ 623 postnatal women: 311 randomly assigned to INT + 312 to CTL	√ 623	√ Mean (SD): 1. INT: 27.5 (5.8); 2. CTL: 28.0 (5.7)	√ Female	No significant differences between INT and CTL
Reid et al., 2002 [103]	√ Randomized controlled trial	√ Evaluate if additional postnatal support in the first PP months could	√ 1004 primiparous women: 753 in INT + 251 in CTL	√ 1004	√ Mean: 26.5	√ Female	Significant group differences in EPDS scores at 6 weeks PP, favoring CTL; no difference at 24 weeks

		influence women's general health					

PP, Postpartum; PPMD, Postpartum major depression; PPD, Postpartum depression; MD, Medical disorders; INT, Intervention group; CTL, Control group; DHA, Docosahexaenoic acid; CPEP, Calcium for Preeclampsia Prevention; WOG, Weeks of gestation; EPDS, Edinburgh Postnatal Depression Scale; LQ, Leverton Questionnaire; CCEI, Crown Crisp Experiential Index; GHQ-D, General Health Questionnaire; PND, Postnatal depression; HMOH, Health Maintenance Organization Hospital.

Supplementary Table S3. Summary of studies investigating postpartum depression preventive interventions using antenatal classes, early postpartum appointments and educational strategies.

Study	Study Design	Aim of the Study	Population	N	Age (Years)	Gender	Results
Webster et al., 2003 [108]	√ Randomized controlled trial	√ Evaluate the effectiveness of an antenatal intervention to reduce PPD	√ 600 pregnant women at risk for PPD: 299 in INT + 301 in CTL	√ 600	√ Mean (SD): 1. INT: 27.8 (6.3); 2. CTL: 26.6 (5.8);	√ Female	No significant differences between INT and CTL
Serwint et al., 1991 [109]	√ Prospective, randomized clinical trial	√ Evaluate if early postnatal appointments may reduce maternal anxiety and depression	√ 251 mothers: 129 in INT + 122 in CTL	√ 251	X	√ Female	No significant differences between INT and CTL
Gunn et al., 1998 [110]	√ Randomized controlled trial	√ Assess if earlier postnatal visits to a GP are effective in reducing PPD rates and improving general maternal wellbeing	√ 683 women giving birth at a rural and a metropolitan hospital	√ 683	√ Mean (SD): 1. INT: 27.5 (5.3); 2. CTL: 28.0 (5.2)	√ Female	No significant differences between the two groups; however, those in INT were more likely to talk about their birth experience, resulting in health gains for women
Okano et al., 1998 [111]	√ Descriptive study	√ Assess the effectiveness of educational strategies provided during pregnancy in reducing PPD	√ 40 Japanese mothers suffering from PPD: 18 in INT + 22 in CTL	√ 40	√ Mean (SD): 1. INT: 25.0 (1.8); 2. CTL: 25.6 (3.1)	√ Female	Educational strategies may decrease PPD severity and the time between the onset of depressive manifestations and access to mental health services
Heh et al., 2003 [112]	√ Quasi-experimental study	√ Assess whether informational support is effective in decreasing PPD severity	√ 70 Taiwanese married women, first-time mothers: 35 in INT + 35 in CTL	√ 70	√ Mean (SD): 1. INT: 26.7 (4.1); 2. CTL: 27.5 (4.3)	√ Female	Informational support on PPD may contribute to reduce the severity of PPD

Hayes et al., 2001 [113]	√ Randomized controlled trial	√ Investigate the effectiveness of antenatal education in preventing PPD	√ 206 primiparous women 28–63 WOG: 103 in INT + 103 in CTL	√ 206	√ Mean: 1. INT: 26.0 [23.0–30.0]; 2. CTL: 25.0 [22.5–29.0]	√ Female	No significant differences between INT and CTL

PPD, Postpartum depression; INT, Intervention group; CTL, Control group; GP, General Practitioner; WOG, Weeks of gestation.

Supplementary Table S4. Summary of studies investigating the impact of marriage on mental health and key elements of healthy marriage promotion.

Study	Study Design	Aim of the Study	Population	N	Age (Years)	Gender	Results
Lewis et al., 2007 [160]	√ 2x2 factorial design	√ Evaluate the role of health-related social control in the promotion of healthy reactions among married individuals	√ 109 couples (218 subjects) being married for 4 years on average	√ 218	√ 18–44 (husbands slightly older than wives)	√ Female/Male	Spouses may monitor one another, encouraging healthy behaviors that promote emotional wellbeing
Gove et al., 1990 [161]	√ Theoretical analysis	X	X	X	X	X	Marriage can create a sense of identity and purpose, contributing to mental wellbeing
Strohschein et al., 2005 [162]	√ Longitudinal study	√ Evaluate gender differences in mental health effects secondary to a marital status change	√ 11155 individuals	√ 11155	√ Mean (SD): 44 (17)	√ Female/Male	Marriage has a positive impact on mental health, while a transition out of marriage is associated with increased psychological distress in both men and women
Simon et al., 2002 [163]	X	√ Revisit the hypothesis according to which the emotional benefits of marriage apply to men more than women	X	X	X	X	Marriage carries emotional benefits for both men and women, however, women report greater psychological distress when their marriages break down
Bianchi et al., 1999 [164]	√ Longitudinal study	√ Evaluate gender differences in the economic situation after marital status change	√ 199 couples who separated	√ 398	X	√ Female/Male	Costs of loss and separation or divorce fall more heavily on women than men

Ooms et al., 2004 [165]	√ Report	√ Provide an introductory discussion on strategies that are effective in the promotion of healthy marriages and reduction of divorce rates	X	X	X	√ Female/Male	Some countries have developed programs aimed at promoting healthy marriages via relationship education as a strategy to improve public health
